# Supplementary material for: Kinetic evidence for multiple aggregation pathways in antibody light chain variable domains
Source: bioRxiv. 2023 Aug 28:2023.08.28.555139. Preprint. [Version 1] doi: 10.1101/2023.08.28.555139 (PMC10491100; doi:10.1101/2023.08.28.555139)
Supplement: Supplement 1 [file NIHPP2023.08.28.555139v1-supplement-1.pdf]

bioRxiv preprint doi: https://doi.org/10.1101/2023.08.28.555139; this version posted August 28, 2023. The copyright holder for this preprint (which was not certified by peer review) is the author/funder, who has granted bioRxiv a license to display the preprint in perpetuity. It is made available under aCC-BY 4.0 International license.

SUPPLEMENTAL FIGURES

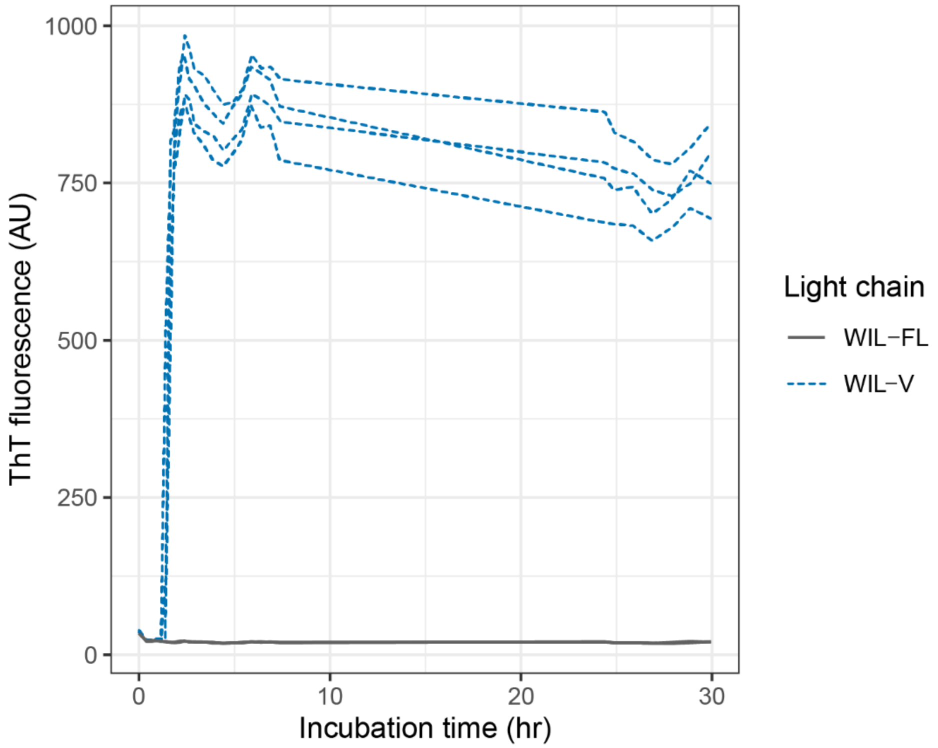

Supplemental figure 1: Full-length WIL remains soluble under conditions where its variable domain aggregates. WIL variants (n=4 wells) were incubated in microplates in PBS, pH 7.4, containing 1 μM ThT at 37 °C and shaken at 500 rpm. Aggregation kinetics were monitored by ThT fluorescence.

bioRxiv preprint doi: https://doi.org/10.1101/2023.08.28.555139; this version posted August 28, 2023. The copyright holder for this preprint (which was not certified by peer review) is the author/funder, who has granted bioRxiv a license to display the preprint in perpetuity. It is made available under aCC-BY 4.0 International license.

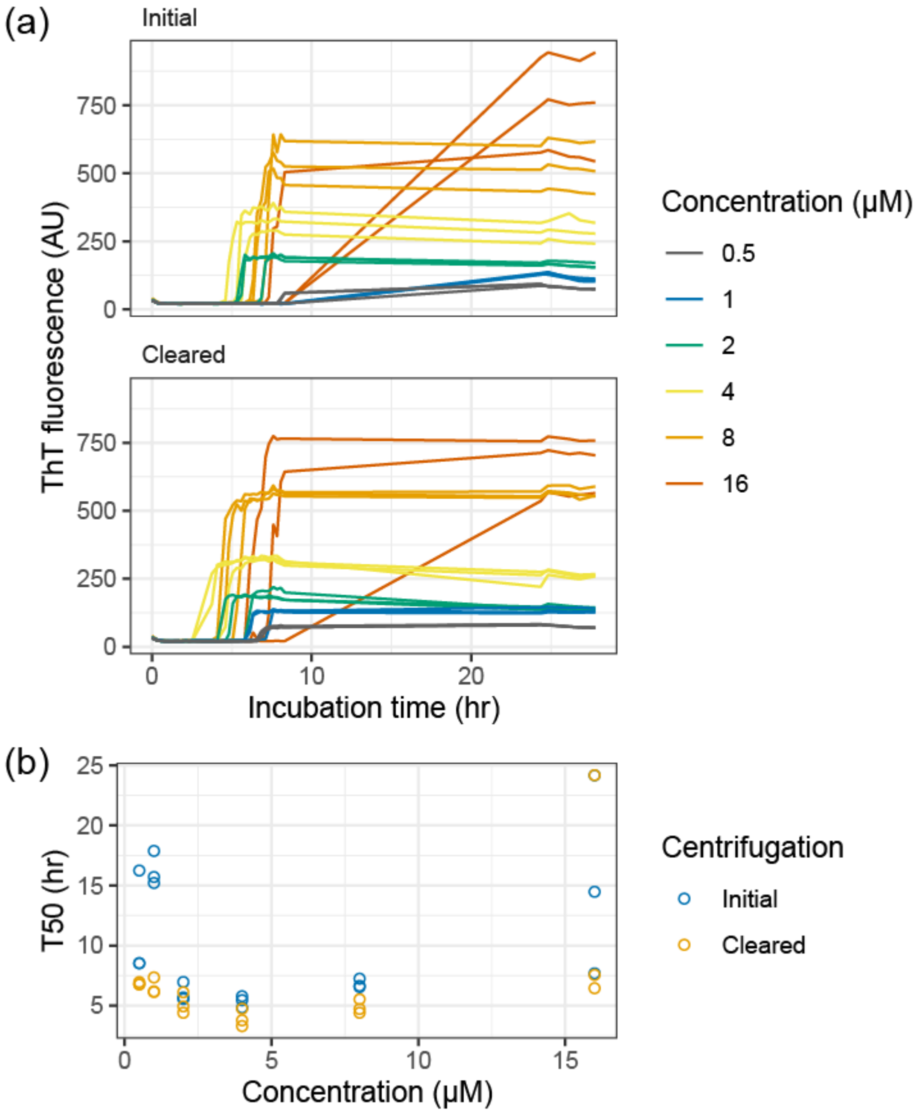

Supplemental figure 2: Pre-clearance of WIL-V-Nhis solutions by ultracentrifugation does not eliminate off-pathway aggregation. WIL-V-Nhis aggregation in PBS, with or without pre-clearance of the stock solution by ultracentrifugation, was monitored on a single microwell plate (n = 3 wells per condition) at 37 °C, 500 rpm. Note that slow aggregation occurred overnight when fluorescence could not be monitored for some samples, which limits the precision of these measurements. (a) ThT fluorescence as a function of time. Colors indicate initial protein concentrations. (b) Calculated midpoint times (T_{50}) for the data shown in (a). Colors indicate pre-treatment.

bioRxiv preprint doi: https://doi.org/10.1101/2023.08.28.555139; this version posted August 28, 2023. The copyright holder for this preprint (which was not certified by peer review) is the author/funder, who has granted bioRxiv a license to display the preprint in perpetuity. It is made available under aCC-BY 4.0 International license.

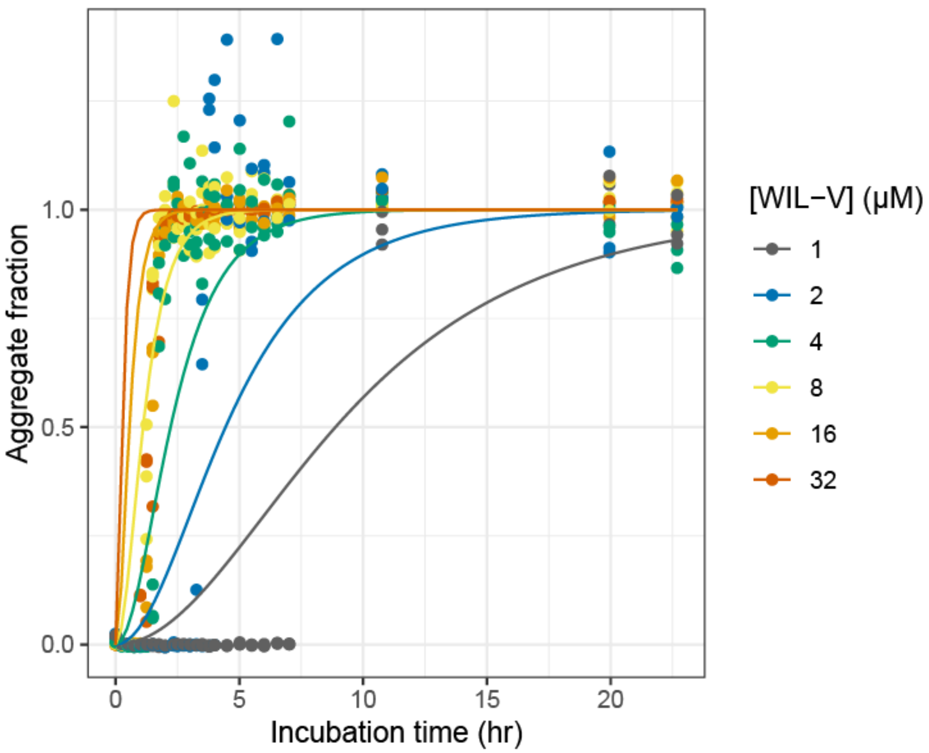

Supplemental figure 3: Existing models do not capture the kinetics of WIL-V aggregation. Example global fit of WIL-V aggregation kinetic data using the AmyloFit program's nucleated polymerization model (Meisl et al., 2016). The model does not capture the long lag phase or rapid extension phase of aggregation, consistent with the hypothesis that more complex processes are involved.

bioRxiv preprint doi: https://doi.org/10.1101/2023.08.28.555139; this version posted August 28, 2023. The copyright holder for this preprint (which was not certified by peer review) is the author/funder, who has granted bioRxiv a license to display the preprint in perpetuity. It is made available under aCC-BY 4.0 International license.

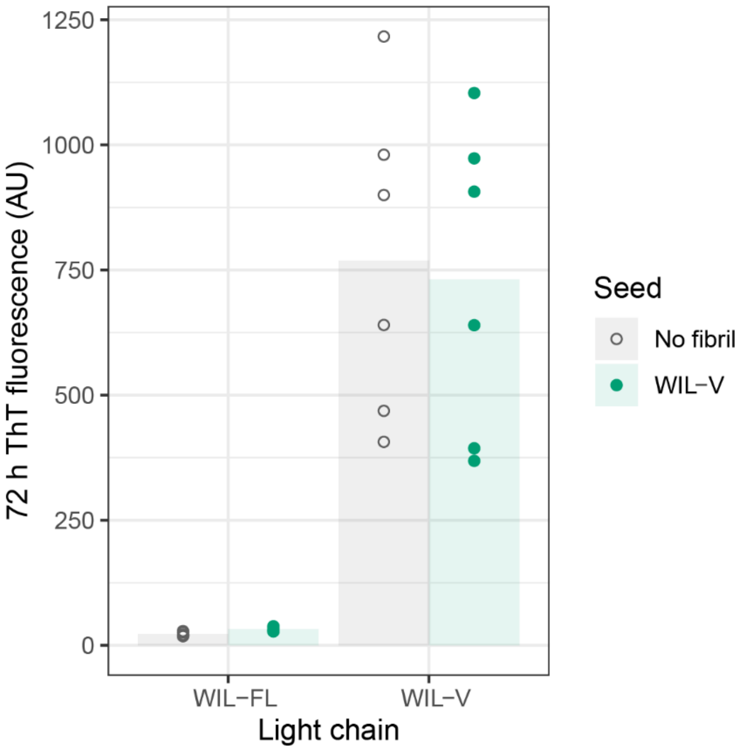

Supplemental Figure 4: Addition of pre-formed fibril seeds does not lead to aggregation of full-length WIL within the timescale of these experiments. The ThT fluorescence observed in WIL-FL solutions (n = 6 wells) did not increase after 72 h incubation (8 μM LC, PBS, pH 7.4, 1 μM ThT, 37 °C, 500 rpm).
